# Optimizing the Probabilistic Neural Network Model with the Improved Manta Ray Foraging Optimization Algorithm to Identify Pressure Fluctuation Signal Features

**DOI:** 10.3390/biomimetics9010032

**Published:** 2024-01-04

**Authors:** Xiyuan Liu, Liying Wang, Hongyan Yan, Qingjiao Cao, Luyao Zhang, Weiguo Zhao

**Affiliations:** 1 School of Water Conservancy and Hydropower, Hebei University of Engineering, Handan 056038, China; jinle16@163.com (X.L.); wangliying@hebeu.edu.cn (L.W.); xianingziwei99@163.com (Q.C.); zly01221214@163.com (L.Z.); 2 Hebei Key Laboratory of Intelligent Water Conservancy, Hebei University of Engineering, Handan 056038, China

**Keywords:** hydraulic turbine, pressure fluctuation, manta ray foraging optimization algorithm, probabilistic neural network, signal identification

## Abstract

To improve the identification accuracy of pressure fluctuation signals in the draft tube of hydraulic turbines, this study proposes an improved manta ray foraging optimization (ITMRFO) algorithm to optimize the identification method of a probabilistic neural network (PNN). Specifically, first, discrete wavelet transform was used to extract features from vibration signals, and then, fuzzy c-means algorithm (FCM) clustering was used to automatically classify the collected information. In order to solve the local optimization problem of the manta ray foraging optimization (MRFO) algorithm, four optimization strategies were proposed. These included optimizing the initial population of the MRFO algorithm based on the elite opposition learning algorithm and using adaptive t distribution to replace its chain factor to optimize individual update strategies and other improvement strategies. The ITMRFO algorithm was compared with three algorithms on 23 test functions to verify its superiority. In order to improve the classification accuracy of the probabilistic neural network (PNN) affected by smoothing factors, an improved manta ray foraging optimization (ITMRFO) algorithm was used to optimize them. An ITMRFO-PNN model was established and compared with the PNN and MRFO-PNN models to evaluate their performance in identifying pressure fluctuation signals in turbine draft tubes. The evaluation indicators include confusion matrix, accuracy, precision, recall rate, F1-score, and accuracy and error rate. The experimental results confirm the correctness and effectiveness of the ITMRFO-PNN model, providing a solid theoretical foundation for identifying pressure fluctuation signals in hydraulic turbine draft tubes.

## 1. Introduction

The problem of vibration occurs during the operation of hydropower units on a regular basis and cannot be avoided [1]. The turbine, which is the most important piece of equipment in a hydropower plant, has a vibration mechanism that is more complicated than that of the other hydropower units. A large number of studies have shown that the operational status of the turbine can be analyzed by classifying and identifying the vibration signal characteristics [2,3] so as to determine whether the turbine needs to be inspected and repaired. Therefore, accurate identification of vibration signals has become particularly important.

The identification of hydraulic turbine vibration signals is a complex problem that involves multiple fields of knowledge. Feature extraction is the first step in identifying the vibration signals of hydraulic turbines. The authors in [4] put forward a method of pressure fluctuation signal analysis based on variational mode decomposition in view of the non-stationarity of the pressure fluctuation signal in the draft tube of a hydraulic turbine. Pan et al. [5] suggested a new approach to analyze hydropower unit vibration data using local mean decomposition (LMD) and Wigner–Ville distribution. Lu et al. [6] used empirical node decomposition (EMD) and index energy to extract the dynamic characteristic information of the draft tube of a hydraulic turbine. Oguejiofor et al. [7] used discrete wavelet transform and principal component analysis to identify the power plant reactor coolant pumps’ vibration signals. Four main component variables are enough to determine normal and pathological vibration signals. Shomaki et al. [8] proposed a new framework for bearing fault detection based on wavelet trans-form and discrete Fourier transform combined with deep learning. Lu et al. [9] suggested an upgraded Hilbert–Huang transform (HHT) approach with energy correlation fluctuation criterion for hydroelectric generating unit vibration signal feature extraction. Wang et al. [10] suggested a Hilbert–Huang transform (HHT)-based motor current signal analysis approach to obtain unstable pump operation features under cavitation situations. From this, it can be seen that there are multiple methods that can be used to extract signal features. This study used discrete wavelet transform for the feature extraction of vibration signals, which is an effective method that is widely used in the field of signal processing.

With the rapid development and widespread application of artificial intelligence, more and more research fields such as pattern identification and fault diagnosis are applying artificial intelligence to handle and solve problems. The deep learning method is used to classify and identify the signals of pressure changes in the draught tube of the Francis turbine. This is carried out to keep the turbine from breaking down or to find the problem quickly if the turbine does break down. Luo et al. [11] developed an adaptive Fisher-based deep convolutional neural network for rolling bearing fault identification. Lan et al. [12] optimized the smoothing factor of a probabilistic neural network (PNN) using the fruit fly optimization algorithm (FOA) and constructed an FOA-PNN model. The FOA-PNN model can predict the operating status of steam turbines in a short amount of time and monitor running faults in real time. Ma et al. [13] proposed an identification method based on singular value decomposition (SVD) and MPSO-SVM. Cao et al. [14] suggested an improved artificial bee colony optimization algorithm (IARO) with adaptive weight adjustment. IARO optimized support vector machine (SVM) to create an identification model to classify and identify vibration signals in different stages. Wang et al. [15] proposed a new convolutional neural network model, MIMTNet. This model has multidimensional signal inputs and multidimensional task outputs, which can improve the generalizability and accuracy of bearing fault diagnosis. Li et al. [16] proposed a one-time NAS fault diagnosis method that can use a supernet to include all candidate networks, train a supernet to evaluate the performance of candidate networks, and apply it to industrial faults. Ravikumar et al. [17] proposed a fault diagnosis model including multi-scale deep residual learning (MDRL-SLSTM) with stacked long short-term memory, which is used to process sequential data in the health prediction task of internal combustion engine transmission. Prosvirin et al. [18] suggested diagnosing rubbing problems of varied intensities using multivariate data and a multivariate one-dimensional convolutional neural network. Ji et al. [19] suggested a two-stage intelligent fault diagnosis system (order-tracking one-dimensional convolutional neural network, OT-1DCNN) to address the problem of fault identification under changing speed settings. Zhou et al. [20] proposed a method for the health diagnosis and early warning of electric motor bearings based on an integrated system of empirical mode decomposition, principal component analysis, and adaptive network fuzzy inference (EEMD-PCA-ANFIS).

Optimization algorithms possess excellent adaptability and global search capabilities [21], which can improve neural networks by improving the way they work and making them more accurate. Sánchez et al. [22] presented a new optimization technique for modular neural network (MNN) architecture based on granular computing and the firefly algorithm. The method was tested to verify its effectiveness and advantages in terms of face identification and compared with other methods. The results show that the method is effective at optimally designing neural networks in pattern identification. Lan et al. [12] suggested optimizing probabilistic neural network (PNN) smoothing factors with the drosophila optimization algorithm (FOA). To categorize the operational condition of hydraulic turbine units, an FOA-PNN network model was constructed. The FOA-PNN has been shown to predict steam turbine operating conditions quickly and monitor operational issues in real time.

Based on the above research, the following conclusions can be drawn: extracting vibration signal features is the first step in signal identification; in the field of fault diagnosis, the accurate identification of vibration signal features is crucial; and optimization algorithms have emerged as a useful tool in neural network training, allowing for the development of more precise and efficient answers to many problems. High-precision signal feature identification technology can quickly and accurately locate fault points, and pressure fluctuation changes can reflect the water turbine’s operating status. Therefore, this study mainly evaluated the feature identification method of the pressure fluctuation signal in the draft tube of a hydraulic turbine.

### 1.1. Motivation and Contribution

The safe and stable operation of hydraulic turbines is crucial for energy production and supply, and vibration signals are one of the important indicators of hydraulic turbine faults and problems. In order to better analyze and apply these signals, we used discrete wavelet transform for the feature extraction of vibration signals, which is an effective method that is widely used in the field of signal processing. By extracting the features, useful information in the vibration signal can be revealed, thereby achieving identification and analysis of the state of the hydraulic turbine.

In addition, in order to improve the accuracy and efficiency of signal classification and identification, optimization algorithms have played an important role. However, the manta ray foraging optimization (MRFO) algorithm often has problems such as being prone to falling into local optima. In order to improve the manta ray foraging optimization (MRFO) algorithm, we selected four improvement strategies. Firstly, by optimizing the initial population of the manta ray foraging optimization algorithm based on the elite-opposition-based learning algorithm, the algorithm’s initial search ability was improved. Secondly, an adaptive t distribution was used to replace the chain factor and optimize individual update strategies and other improvement strategies to improve the algorithm’s global search ability. These improvement strategies helped to improve the optimization performance of the manta ray foraging optimization algorithm. And by comparing the performance with particle swarm optimization (PSO), the sparrow search (SSA) algorithm, and the manta ray foraging optimization (MRFO) algorithm on 23 test functions, the superiority of the improved manta ray foraging optimization (ITMRFO) algorithm was verified.

Neural networks are commonly used for signal identification and pattern classification tasks. Probabilistic neural networks (PNNs) are particularly popular because they have a fast-learning speed and can be directly trained. When using probabilistic neural networks for classification, the smoothing factor is a key input parameter that affects the smoothness of decision boundaries. The selection of smoothing factors directly affects the performance and classification results of probabilistic neural networks. In order to optimize the smoothing factor of the probabilistic neural network, we adopted the improved manta ray foraging optimization (ITMRFO) algorithm for training. By optimizing the improved manta ray foraging optimization (ITMRFO) algorithm, we could determine the optimal smoothing factor, thereby improving the classification accuracy of probabilistic neural network (PNN) feature identification.

The main contributions of this study are highlighted as follows.

- Discrete wavelet transforms extracts pressure fluctuation signal features. The fuzzy c-means (FCM) clustering algorithm method, a partition-based clustering algorithm, classifies extracted features and automatically classifies vibration signal characteristics.
- To improve the manta ray foraging optimization (MRFO) algorithm, four improvement methodologies were chosen, including an elite-opposition-based learning algorithm and adaptive t distribution. The improved manta ray foraging optimization (ITMRFO) algorithm method was put to the test using 23 benchmark functions. The experimental findings demonstrate the good exploration and exploitation capabilities of the ITMRFO algorithm.
- Experiments were carried out on the vibration signal of the draft tube of a mixed flow turbine, and verification was achieved through the confusion matrix, accuracy, precision, recall, F1-score, and identification error rate of the training samples. The usefulness of the probabilistic neural network (PNN) identification method optimized by the improved manta ray foraging optimization (ITMRFO) algorithm was demonstrated. The results of the experiments show that the ITMRFO-PNN model is effective at identifying the vibration signs of the draft tube of the hydraulic turbine.

### 1.2. Paper Organization

The residual section of this study is as follows. Section 2 introduces the basic concepts of discrete wavelet transform, the fuzzy c-means (FCM) clustering algorithm, the manta ray foraging optimization (MRFO) algorithm, and the probabilistic neural network (PNN). The MRFO algorithm was improved, producing the ITMRFO algorithm. The improved manta ray foraging optimization (ITMRFO) algorithm was tested using 23 test functions, and the basic overview of the ITMRFO-PNN model was introduced. The identification results of the PNN model, the MRFO-PNN model, and the ITMRFO-PNN model were compared. The effectiveness and accuracy of the ITMRFO-PNN model were verified by comparing these three models through a confusion matrix, accuracy, precision, recall rate, and F1-score. Section 4 introduces the conclusions and future development directions.

## 2. Materials and Methods

### 2.1. Discrete Wavelet Transform

**Figure 1:**
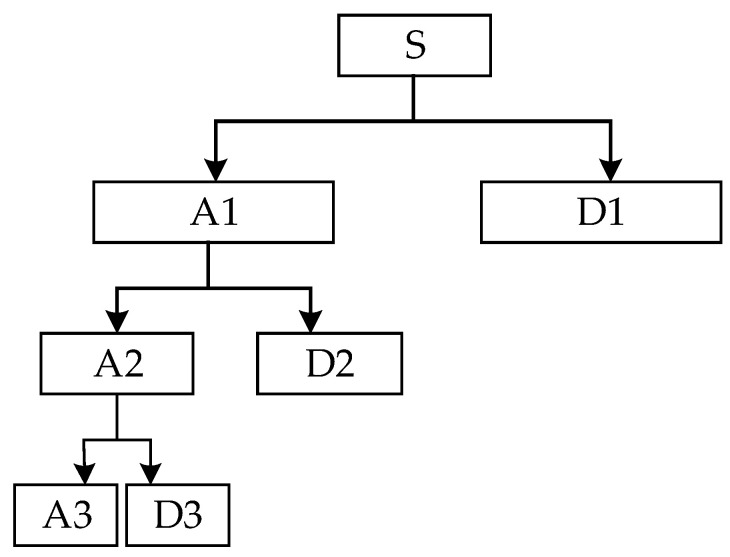
The schematic diagram of wavelet analysis.

In the 1980s, French scientists Grossman and Morlet proposed wavelet analysis for the analysis of seismic signals. Wavelet analysis is a method for analyzing non-stationary signals. The schematic diagram of wavelet analysis is shown in Figure 1.

Discrete wavelet transform (DWT) [23] is an important time-frequency analysis method, which can decompose nonlinear and non-stationary signals at multiple scales. Discrete wavelet transform decomposes the signal into a series of scale coefficients (approximate coefficients) and wavelet coefficients (detail coefficients). The wavelet inverse transform can effectively extract useful information from the signal by selecting appropriate coefficients. The basis function of the discrete wavelet is:

(1) $$WT_{s}\left(s,k\right)=\langle x\left(t\right),\psi_{s,k}(t)\rangle$$

### 2.2. Fuzzy C-Means Clustering Algorithm

Dumm proposed the fuzzy c-means clustering (FCM) [24] algorithm in 1974, and Bezdek improved and extended it in 1981. FCM clustering is to take a given set of samples $X=\left\{x_{1},x_{2}\ldots x_{n}\right\}$, divide this n-sample set into c classes, where 2≤c<n, based on certain clustering principles and fuzzy criteria, and denote this c class by $Y=\left\{y_{1},y_{2}\ldots y_{c}\right\}$. The FCM algorithm has the following objective function:

(2) $$J_{FCM}\left(U,Y\right)=\sum_{i=0}^{n}\sum_{j=1}^{c}u_{ij}^{m}{∥x_{i}-y_{j}∥}^{2}(m\geq 0)$$

where c is the number of clusters, n is the data sample, $\sum_{i=1}^{c}u_{ij}=1,u_{ij}\in [0,1]$ is the weighting index $\left(m\in [0,\infty )\right)$, and U is the input space X a fuzzy c-ization score. The Euclidean distance between the i-th sample data $x_{i}$ and the j-th clustering center $y_{j}$ is ${∥x_{i}-y_{j}∥}^{2}$.

The fuzzy c-means (FCM) clustering algorithm technique is frequently used [25,26] to determine the class of the sample points for the goal of automatically categorizing the sample data by obtaining the affiliation of each sample point to all class centers by optimizing the objective function. Therefore, the fuzzy c-means (FCM) clustering algorithm method is used to group the extracted pressure fluctuation eigenvectors.

### 2.3. An Improved MRFO Algorithm

#### 2.3.1. MRFO Algorithm

In 2020, Zhao W. et al. [27] proposed a novel intelligent bionic population method called the manta ray foraging optimization algorithm.

The MRFO algorithm is a mathematical modeling of the foraging process of manta rays in the ocean, and it is based on the three foraging strategies of manta rays. Compared to intelligent heuristics such as particle swarm optimization and simulated annealing, the MRFO algorithm is characterized by high merit-seeking ability, fast convergence, and few parameters [28,29,30].

1. Chain foraging
  (3) $$x_{i}^{d}\left(t+1\right)=\left\{\begin{array}{c} x_{i}^{d}\left(t\right)+r(x_{best}^{d}\left(t\right)-x_{i}^{d}\left(t\right))+\alpha (x_{best}^{d}-x_{i}^{d}\left(t\right)),i=1\\ x_{i}^{d}\left(t\right)+r(x_{i-1}^{d}\left(t\right)-x_{i}^{d}\left(t\right))+\alpha (x_{best}^{d}-x_{i}^{d}\left(t\right)),i=2,\ldots ,N \end{array}\right.$$
  (4) $$\alpha =2r\sqrt{\left|\log r\right|}$$
  where $x_{i}^{d}\left(t\right)$ denotes the position of the tth generation, the ith individual in the dimension, r denotes a random number uniformly distributed on [0,1], $x_{best}^{d}\left(t\right)$ denotes the position of the best individual in the tth generation in the dth dimension, N denotes the number of individuals, and α is the chain factor.
2. Spiral foraging
  When $\frac{t}{T}>rand$
  (5) $$x_{i}^{d}\left(t+1\right)=\left\{\begin{array}{c} x_{best}^{d}\left(t\right)+r(x_{best}^{d}\left(t\right)-x_{i}^{d}\left(t\right))+\beta (x_{best}^{d}-x_{i}^{d}\left(t\right)),i=1\\ x_{best}^{d}\left(t\right)+r(x_{i-1}^{d}\left(t\right)-x_{i}^{d}\left(t\right))+\beta (x_{best}^{d}-x_{i}^{d}\left(t\right)),i=2,\ldots ,N \end{array}\right.$$
  (6) $$\beta =2e^{r_{1}\frac{T-i+1}{T}}sin\left(2\pi r_{1}\right)$$
  where T is the total number of iterations, $r_{1}$ is a random number evenly distributed on the range [0,1], and β is the spiral factor.
  When $\frac{t}{T}\leq rand$
  (7) $$x_{i}^{d}\left(t+1\right)=\left\{\begin{array}{c} x_{rand}^{d}\left(t\right)+r(x_{rand}^{d}\left(t\right)-x_{i}^{d}\left(t\right))+\beta (x_{rand}^{d}-x_{i}^{d}\left(t\right)),i=1\\ x_{rand}^{d}\left(t\right)+r(x_{i-1}^{d}\left(t\right)-x_{i}^{d}\left(t\right))+\beta (x_{rand}^{d}-x_{i}^{d}\left(t\right)),i=2,\ldots ,N \end{array}\right.$$
  (8) $$x_{rand}^{d}=Lb^{d}+r\left(Ub^{d}-Lb^{d}\right)$$
  where $x_{rand}^{d}\left(t\right)$ indicates the random position in generation tth and dimension dth. The upper and lower limits of a variable are denoted by $Ub^{d}\;and\;Lb^{d}$.
3. The rolling foraging
  (9) $$x_{i}^{d}\left(t+1\right)=x_{i}^{d}\left(t\right)+S\left(r_{2}x_{best}^{d}-r_{3}x_{i}^{d}\left(t\right)\right),\;i=1,2,\ldots ,N$$
  (10) S=2
  where S is the rollover factor, and the random integers $r_{2}$ and $r_{3}$ are both equally distributed on the range [0,1].

The MRFO algorithm’s rapid convergence and prematurity lead to poor population diversity, making it easy to slip into local optimal solutions [31]. To increase population variety and jump out of the local optimum, the initial population of the MRFO algorithm, chain foraging strategy, and spiral foraging strategy are all modified.

#### 2.3.2. Elite Opposition-Based Learning

The population intelligent optimization algorithm’s performance is greatly influenced by the quality of the starting population, and a high-quality starting population increases the likelihood of finding the best overall solution. Tizhoosh [32] proposed the opposition-based learning strategy (OBL) in 2005, which has been shown to increase the probability of finding a globally optimum solution by almost 50% [33] and has been applied to various optimization algorithms [34,35,36]. The main idea behind elite-opposition-based learning is to use the practical information of elite individuals to create opposite individuals in the area where the current individuals are. The opposite individuals are then placed in competition with the current individuals, and the best individuals are chosen to be the next generation.

The elite opposition-based learning strategy is used in the MRFO initialization phase to construct the opposite population of the current population. In order to create a new population, the original population is mixed with the opposing population. The new population is ranked according to population quality. In order to ensure the quality of the selected population, the first 50% of the new population is selected as the initial solution.

#### 2.3.3. Adaptive T-Distribution Strategy

T-distributions, also known as student distributions, contain parametric degrees of freedom. The shape of the curve of the t-distribution is related to the size. The lesser the degree of freedom, the flatter the t-distribution curve, the lower the center of the curve, and the bigger the tail on both sides [37,38]. The probability density is as follows:

(11) $$p(x)=\frac{\Gamma \left(\frac{n+1}{2}\right)}{\sqrt{n\pi }\Gamma (\frac{n}{2})}\cdot {\left(1+\frac{x^{2}}{n}\right)}^{-\frac{n+1}{2}}$$

where $\Gamma \left(\frac{n+1}{2}\right)=\int_{0}^{+\infty }x^{\frac{k+1}{2}-1}e^{-x}\mathrm{dx}$ is the Euler integral of the second kind, Γ() denotes the gamma function. n represents the degrees of freedom parameter for the t-distribution. x is the value at which the probability density function is evaluated.

**Figure 2:**
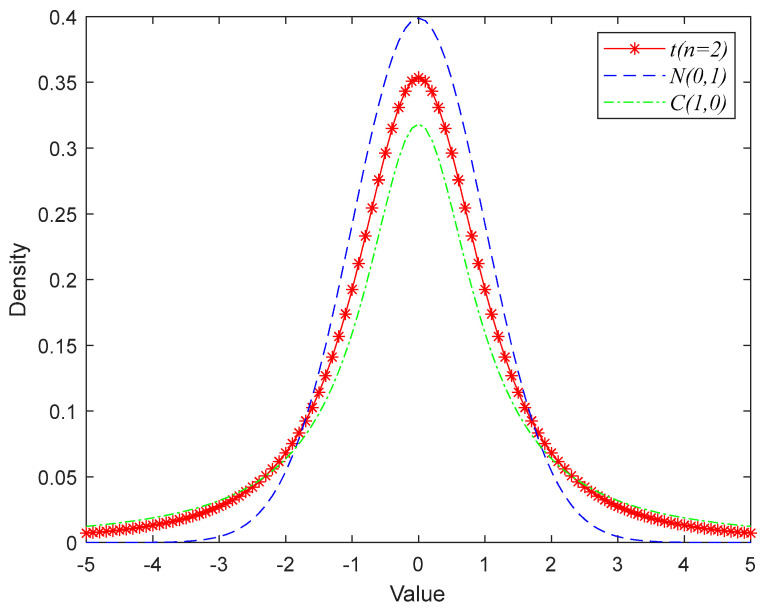
Probability density plot of Cauchy distribution, Gaussian distribution, and T-distribution.

When t(n→∞)→N(0,1), both deviations can often be disregarded for n≥30. However, when $t\left(n=1\right)=C(0,1)$, the Cauchy distribution is C(0,1) and the Gaussian distribution is N(0,1). In other words, the two boundary special case distributions of the t-distribution are the standard Gaussian distribution and the Cauchy distribution. The relationship between the densities of the Gaussian, Cauchy, and t distributions is shown in Figure 2. The tail curve of the Cauchy distribution has a long and flat form, while the tail curve of the normal distribution has a short and steep form. And the Cauchy variation has a greater likelihood of producing next-generation points far from the parent than the Gaussian variation.

In the chain predation process, manta rays form a predation chain from head to tail. The moving direction and step length of the next position of individual manta rays are jointly determined by the current optimum solution and the part of the previous individual. T-distribution is used to replace the chain factor α in the chain foraging place of the MRFO algorithm.

In the chain foraging and spiral foraging of the MRFO algorithm, r is stochastic and unstable, so the part of the equation that multiplies with r is removed. The improved algorithm is named ITMRFO.

#### 2.3.4. ITMFRO Algorithm

1. Chain foraging
  (12) $$x_{i}^{d}\left(t+1\right)=\left\{\begin{array}{c} x_{i}^{d}\left(t\right)+t(x_{best}^{d}-x_{i}^{d}\left(t\right)),i=1\\ x_{i}^{d}\left(t\right)+t(x_{best}^{d}-x_{i}^{d}\left(t\right)),i=2,\ldots ,N \end{array}\right.$$
  where $x_{i}^{d}\left(t\right)$ denotes the position of the tth generation, the ith individual in the dimension, $x_{best}^{d}\left(t\right)$ denotes the position of the best individual in the tth generation in the dth dimension, N denotes the number of individuals, and t is the t-distribution.
2. Spiral foraging
  When $\frac{t}{T}>rand$
  (13) $$x_{i}^{d}\left(t+1\right)=\left\{\begin{array}{c} x_{best}^{d}\left(t\right)+\beta (x_{best}^{d}-x_{i}^{d}\left(t\right)),i=1\\ x_{best}^{d}\left(t\right)+\beta (x_{best}^{d}-x_{i}^{d}\left(t\right)),i=2,\ldots ,N \end{array}\right.$$
  (14) $$\beta =2e^{r_{1}\frac{T-i+1}{T}}sin\left(2\pi r_{1}\right)$$
  where T is the total number of iterations, $r_{1}$ is a random number evenly distributed on the range [0,1], and β is the spiral factor.
  When $\frac{t}{T}\leq rand$
  (15) $$x_{i}^{d}\left(t+1\right)=\left\{\begin{array}{c} x_{k}^{d}\left(t\right)+\beta (x_{rand}^{d}-x_{i}^{d}\left(t\right)),i=1\\ x_{k}^{d}\left(t\right)+\beta (x_{rand}^{d}-x_{i}^{d}\left(t\right)),i=2,\ldots ,N \end{array}\right.$$
  (16) $$x_{rand}^{d}=Lb^{d}+r\left(Ub^{d}-Lb^{d}\right)$$
  where $x_{rand}^{d}\left(t\right)$ indicates the random position in generation tth and dimension dth and $x_{k}^{d}\left(t\right)$ indicates the top 50% of the population.
3. The rolling foraging
  (17) $$x_{i}^{d}\left(t+1\right)=x_{i}^{d}\left(t\right)+S\left(r_{2}x_{best}^{d}-r_{3}x_{i}^{d}\left(t\right)\right),\;i=1,2,\ldots ,N$$
  (18) S=2
  where S is the rollover factor and the random integers $r_{2}$ and $r_{3}$ are both equally distributed on the range [0,1].

#### 2.3.5. Algorithm Comparison Validation

There were 23 chosen benchmark test functions [39]. Experimental comparisons were made between numerical simulations of four intelligence algorithms, including the sparrow search algorithm (SSA), the particle swarm optimization (PSO) algorithm, the manta ray foraging optimization (MRFO) algorithm, and the improved manta ray foraging optimization (ITMRFO) algorithm. Based on the optimum value, the worst value, the mean value, and the standard deviation, these four algorithms were assessed.

For this experiment, a maximum of 500 iterations were allowed, and the initial population size for each of the four algorithms was set at 30. The setting parameters of these four algorithms can be seen in Table 1. Table 2, Table 3, and Table 4 show the unimodal test function, multimodal test function, and fixed-dimension multimodal test function, respectively.

As can be seen in Table 5, the sparrow search algorithm (SSA) obtains the optimal global solutions for the optimum in functions F1–F5, and it obtains the optimal global solutions for the optimum, worst, mean, and standard deviation in function F5. The manta ray foraging optimization (MRFO) algorithm obtains globally optimal solutions in functions F2 and F4 for the standard deviation, and it also obtains globally optimal solutions in functions F1, F3, and F6 for the optimum, worst, mean, and standard deviation. The improved manta ray foraging optimization (ITMRFO) algorithm obtains globally optimal solutions in functions F1–F3 and F7 for the optimum, worst, mean, and standard deviation. And it obtains the optimal global solutions for the worst, mean, and standard deviation in function F4.

**Figure 3:**
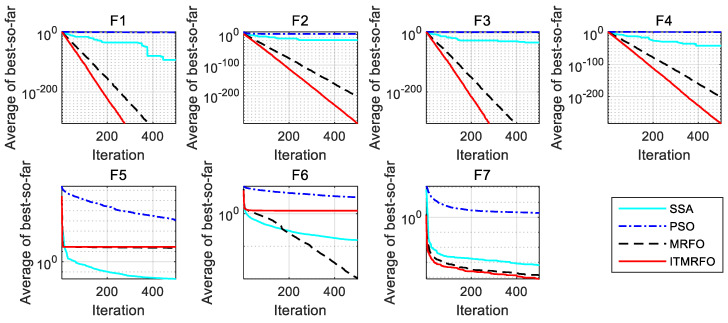
Convergence characteristics of the four algorithms on unimodal test functions.

As can be seen in Figure 3, the sparrow search algorithm (SSA) in function F5 has higher convergence accuracy than the other three algorithms. However, the manta ray foraging optimization (MRFO) algorithm and the improved manta ray foraging optimization (ITMRFO) algorithm converge faster and more consistently. Furthermore, in function F6, the manta ray foraging optimization (MRFO) algorithm converges faster than the three different algorithms, especially before 200 iterations. Compared to the three different optimization algorithms, the improved manta ray foraging optimization (ITMRFO) algorithm has a faster convergence speed and higher convergence accuracy for functions F1–F4 and F7.

From the above analysis, it can be seen that the convergence curve of particle swarm optimization (PSO) is at a standstill, the optimization is basically stopped, and the local optimization cannot be skipped. Among the four algorithms, the ability to jump out of the local optimum is the worst. The convergence curve of the sparrow search (SSA) algorithm is mainly stagnant, and only a small fraction can jump out of the local optimum. The ability of the sparrow search (SSA) algorithm to jump out of the local optimum is only stronger than that of particle swarm optimization (PSO). Although the manta ray foraging optimization (MRFO) algorithm has a strong ability to jump out of the local optimum, its convergence speed and accuracy are lower than the improved manta ray foraging optimization (ITMRFO) algorithm. Compared with the other three algorithms, the improved manta ray foraging optimization (ITMRFO) algorithm has the advantages of superior performance, high accuracy, and fast convergence speed when dealing with unimodal test functions.

As can be seen in Table 6, the sparrow search algorithm (SSA) obtains the glob-ally optimal solutions of the optimum, worst, mean, and standard deviation in functions F9–F11 and F13. The manta ray foraging optimization (MRFO) algorithm obtains the optimal global solution of the worst value and standard deviation in function F8 and the optimal global solution of the optimum, worst, mean, and standard deviation in functions F9–F12. The improved manta ray foraging optimization (ITMRFO) algorithm obtains the globally optimal solutions of the optimal value and the mean value in function F8; it also obtains the globally optimal solutions of the optimum, worst, mean, and standard deviation in functions F9–F11.

**Figure 4:**
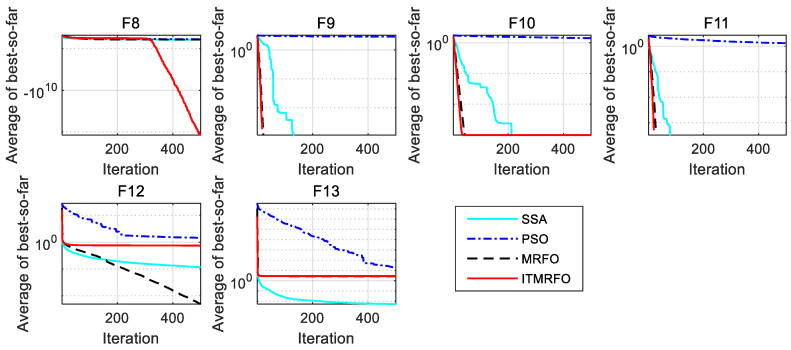
Convergence characteristics of the four algorithms on multimodal test functions.

As can be seen in Figure 4, the sparrow search algorithm (SSA) can jump out of the local optimum and converge more accurately than the other three algorithms in function F13. Still, the manta ray foraging optimization (MRFO) algorithm and improved manta ray foraging optimization (ITMRFO) algorithm can also jump out of the local optimum and are more stable than the SSA. The manta ray foraging optimization (MRFO) algorithm starts to converge faster than the other three algorithms before 200 iterations in function F12. Still, the improved manta ray foraging optimization (ITMRFO) algorithm can also jump out of the local optimum and is more stable than the sparrow search algorithm (SSA) and the manta ray foraging optimization (MRFO) algorithm. The improved manta ray foraging optimization (ITMRFO) algorithm converges fastest in functions F9–F11 and starts to converge faster and more accurately before 400 iterations in function F8.

From the above analysis, it can be seen that the convergence curve of particle swarm optimization (PSO) is mostly in a stagnant state, and the ability to jump out of the local optimum is the worst among the four algorithms. Although the sparrow search algorithm (SSA) has a strong ability to jump out of the local optimum, its convergence speed is slow. Although the manta ray foraging optimization (MRFO) algorithm has a strong ability to jump out of the local optimum, its convergence speed is lower than the improved manta ray foraging optimization (ITMRFO) algorithm. Therefore, the improved manta ray foraging optimization (ITMRFO) algorithm has the best performance, high convergence accuracy, and most of the fastest convergence speeds when dealing with multimodal test functions.

According to Table 7, the sparrow search algorithm (SSA) obtains globally optimal solutions for the optimum, worst, mean, and standard deviation in functions F21–F23. The sparrow search algorithm (SSA) obtains globally optimal solutions for the optimum in functions F14–F15 and F17–F20. The sparrow search algorithm (SSA) obtains globally optimal solutions for the worst and mean in functions F15–F19. The sparrow search algorithm (SSA) obtains globally optimal solutions for the standard deviation in function F15. Particle swarm optimization (PSO) obtains globally optimal solutions for the optimum in functions F14, F16–F19, and F21–F23, and it also obtains globally optimal solutions for the worst and mean in functions F14 and F16–F17. The manta ray foraging optimization (MRFO) algorithm obtains globally optimal solutions for the optimum, worst, mean, and standard deviation in functions F14, F17, and F19. The manta ray foraging optimization (MRFO) algorithm obtains globally optimal solutions for the optimum in functions F16, F18, and F23. The manta ray foraging optimization (MRFO) algorithm obtains globally optimal solutions for the worst and mean in functions F16, F18, and F20. The manta ray foraging optimization (MRFO) algorithm obtains globally optimal solutions for the standard deviation in function F20. The improved manta ray foraging optimization (ITMRFO) algorithm obtains globally optimal solutions for the optimum, worst, mean, and standard deviation in functions F16–F18. The improved manta ray foraging optimization (ITMRFO) algorithm obtains globally optimal solutions for the optimum in functions F14–F15, F19, and F21–23. The improved manta ray foraging optimization (ITMRFO) algorithm obtains globally optimal solutions for the worst in function F19. The improved manta ray foraging optimization (ITMRFO) algorithm obtains globally optimal solutions for the worst in functions F19–F20. The improved manta ray foraging optimization (ITMRFO) algorithm obtains globally optimal solutions for the mean in functions F19–F20.

**Figure 5:**
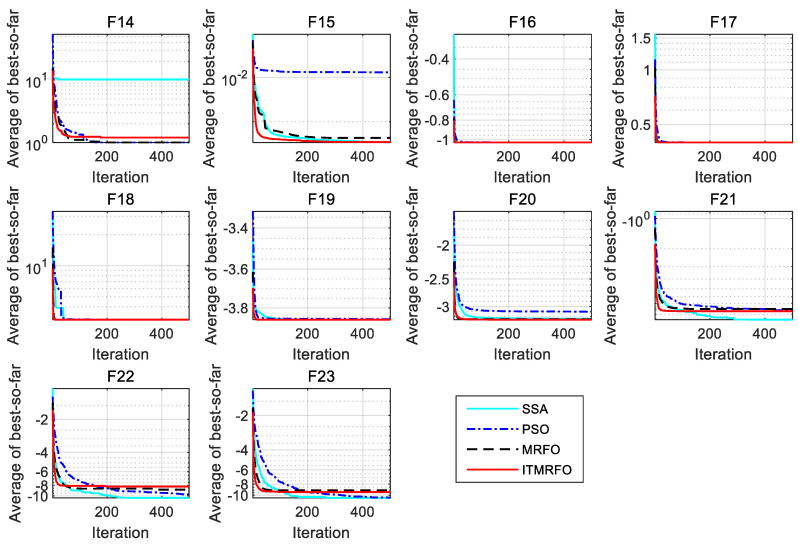
Convergence characteristics of the four algorithms on fixed-dimensional multimodal test functions.

**Figure 6:**
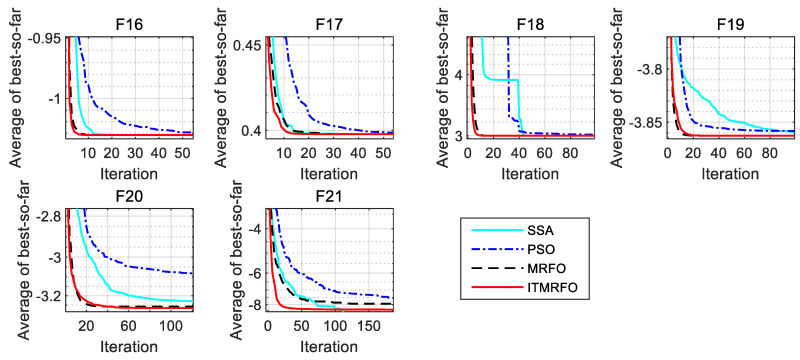
Partial enlargement of F16-F21.

From Figure 5 and Figure 6, it can be seen that the sparrow search algorithm (SSA) converges with higher accuracy on functions F21–F23 than the other three algorithms. The manta ray foraging optimization (MRFO) algorithm converges with higher accuracy on functions F14, F17, and F19. The improved manta ray foraging optimization (ITMRFO) algorithm converges faster and with higher accuracy on functions F15–F18 and F21. In addition, the ITMRFO algorithm converges faster and more consistently than the sparrow search algorithm (SSA).

The above analysis shows that all four intelligent optimization algorithms have better optimization results in fixed-dimensional multimodal test functions. Still, the improved manta ray foraging optimization (ITMRFO) algorithm has superior performance in dealing with fixed-dimensional multimodal test functions, with high convergence accuracy and mostly the fastest convergence speed.

Based on the optimization results of the four intelligent algorithms in 23 bench-mark test functions, it can be concluded that the improved manta ray foraging optimization (ITMRFO) algorithm is faster in terms of convergence speed and has higher stability and accuracy of convergence.

### 2.4. Probabilistic Neural Networks

**Figure 7:**
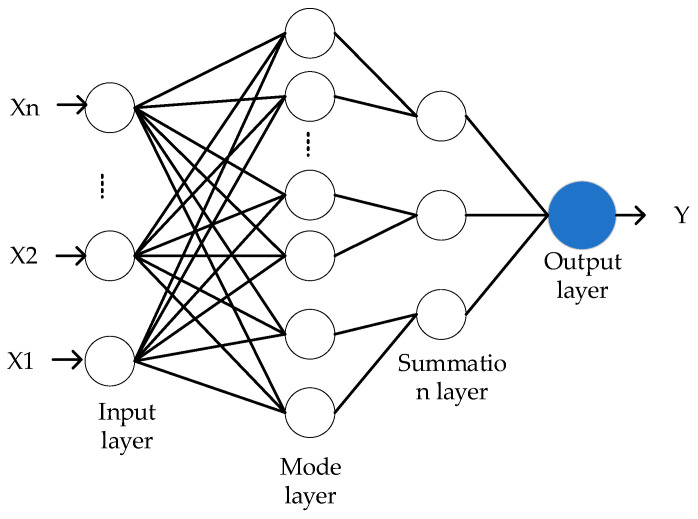
Basic structure of the PNN.

The RBF [40] neural network, a feed-forward network based on the rule of Bayes, was developed by Dr. D.F. Speeht in 1989 [41]. The probabilistic neural network (PNN) is a variation of this network. Probabilistic neural networks (PNNs) are quick to learn, simple to train, and highly accurate in classifying objects. The global optimal solution probabilistic neural network (PNN) consists of the input layer, modal layer, accumulation layer, and output layer. The schematic diagram of the probabilistic neural network (PNN) is shown in Figure 7.

The input layer simply feeds eigenvectors into the neural network without performing any calculations.

The output of each pattern unit is determined as the pattern layer computes the matching relationship between each pattern in the training set using the eigenvectors from the input layer.

(19) $$P(X,W_{a})=exp[-\frac{{\left(X-W_{a}\right)}^{T}\left(X-W_{a}\right)}{2\delta^{2}}]$$

The variable $W_{a}$ is the identification sample, which also serves as the weight of the probabilistic neural network (PNN) connecting the input values and modalities; a is the number of training samples; and the smoothing factor, δ, significantly affects the performance of the probabilistic neural network (PNN).

The summation layer sums and averages the output of neurons of the same class in the pattern layer, which is expressed as:

(20) $$f(x)=\frac{\sum_{a=1}^{N}P(X,W_{a})}{N}$$

The output layer, which accepts the output of the summation layer, is made up of competing neurons. It performs a direct threshold discrimination, and eventually determines the category that corresponds to the characteristic line vector. It is written as:

(21) Y=argmaxf(x)

### 2.5. ITMRFO-Based Optimization of PNN Networks

Traditional neural networks need to be trained on a large amount of data due to their complex structure, which hinders their capacity to generalize and make accurate predictions. In contrast, probabilistic neural network (PNN) models use fewer parameters, do not require initial weight setting, and reduce human subjectivities affecting the model parameters and randomness in the network architecture.

The probabilistic neural network is a parallel method that uses the Bayesian minimum risk criterion to solve pattern classification problems [42,43]. A probabilistic neural network is “trained” using input training samples to establish the network’s size, neuron centroids, connection thresholds, and weights. The threshold determines the width of the radial basis function. The larger the threshold, the greater the decay of the radial basis function as the input vector moves away from the weight vector, which affects the accuracy of the probabilistic neural network model. The probabilistic neural network (PNN) estimates the smoothing factor δ based on the minimum aver-age distance between samples. As a result, identical data points will bias the smoothing factor estimate, affecting the operating state and accuracy of the probabilistic neural network (PNN) model. The smoothing factor automatically adjusts the threshold of the pattern layer. Therefore, only the smoothing factor needs to be optimized.

**Figure 8:**
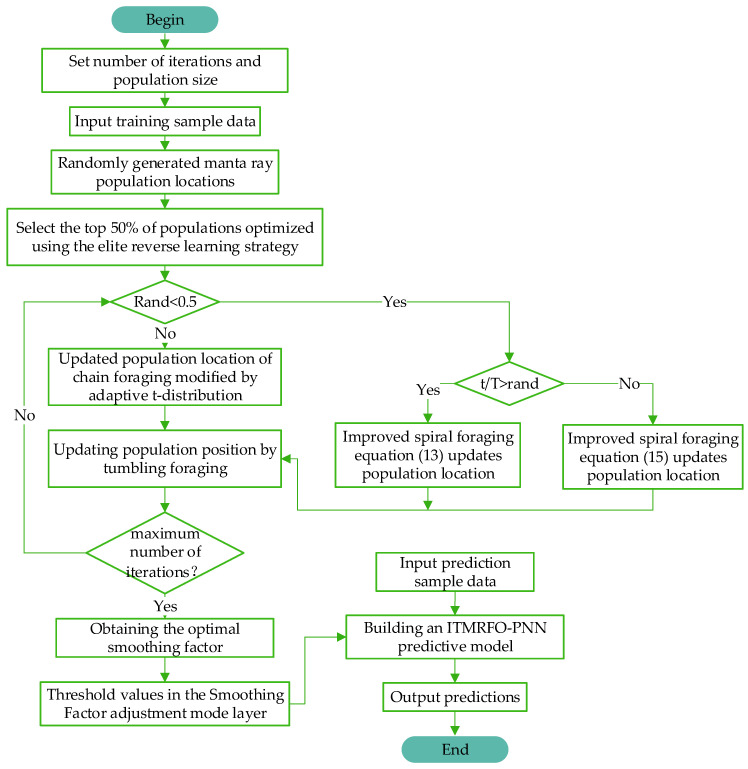
Basic flow chart of the ITMRFO-PNN.

The improved manta ray foraging optimization (ITMRFO) algorithm is used to optimize the smoothing factor of the probabilistic neural network (PNN). The ITMR-FO-PNN flow chart is shown in Figure 8.

The workflow for the ITMRFO-PNN model is as follows: (1) input the training sample data; (2) establish the population and specify the manta ray parameters (select the best population with the elite-opposition-based algorithm); (3) determine Rand < 0.5. If it holds, perform spiral foraging. Perform chain foraging if it does not hold; (4) calculate the fitness value and update the optimum position; (5) perform rollover foraging and update the position. (6) calculate the fitness value and update the optimum position; and (7) judge whether the end condition is satisfied; if so, output the optimum value; otherwise repeat steps 2–7.

### 2.6. Model Structure

The eigenvectors of draft tube pressure fluctuation are extracted by wavelet analysis, and the fuzzy c-means (FCM) clustering algorithm classifies the extracted eigenvectors. The probabilistic neural network (PNN) has the advantages of a simple structure, fast training speed, strong classification ability, etc. By training the vibration characteristic vector of draft tube pressure fluctuation, the probabilistic neural network (PNN) model is established to analyze the draft tube pressure fluctuation. The threshold and smoothing factor also impact how accurately the probabilistic neural network (PNN) can classify data. In this study, the smoothing factor of the probabilistic neural network (PNN) was optimized by the improved manta ray foraging optimization (ITMRFO) algorithm. The optimized smoothing factor automatically modifies the threshold. The ITMRFO-PNN network model with more accurate classification results was established. The pressure fluctuation analysis model of the draft tube of the hydraulic turbine was established.

## 3. Experimental Process

### 3.1. Analysis of Experimental Data

The total installed capacity of the hydropower station considered is 1000 MW, and it is equipped with four mixed flow turbines with a single unit capacity of 250 MW. The rated head of the water turbine at the hydropower station is 305 m, and the rated speed is 333.3 r/min. In the hydropower station, the four turbines are of the same type and are in normal operation. This study classified and identified the characteristics of the pressure fluctuation signal of the draft tube of four normal running turbines. The sensor type was the INV9828 piezoelectric acceleration sensor, and the sampling frequency of the vibration signal was 1024 Hz.

The vibration signal acquisition process is shown below:

- Vibration measurement points were set up at the inlet and outlet of the draft tube of Units 1–4 of the hydropower station.
- The data measured at the inlet and outlet vibration points of the same unit were combined into a group of data. The pressure fluctuation data of four units in three different time periods was recorded during water pumping, and the pressure fluctuation data of four units in three different time periods were recorded during power generation.
- Finally, the pressure fluctuation data of the draft tube of these four units (24 sets of data in total) were analyzed.

The twenty-four groups of test data can be seen in Table 8. W1 represents the pumping time of Unit 1, and P1 indicates when Unit 1 generates power. The draft tube pressure fluctuation data for Unit 1 (number 11A) are shown in Table 9.

### 3.2. Processing of Hydraulic Turbine Vibrations

There are 24 sets of data, each containing vibration data measured at two vibration measurement points. Firstly, we adopted a classic fixed threshold denoising method that is widely used in the field of signal processing. Compared with adaptive threshold methods, fixed threshold methods use predetermined threshold values to denoise signals, simplifying the process of threshold selection. At this stage, we used the Coif4 wavelet from the Coiflet wavelet series as the wavelet basis function. The Coif4 wavelet is a wavelet basis function with order 4 in the Coiflet series, which exhibits good characteristics in both the time and frequency domains. This combination can effectively denoise signals and capture their local features and detailed information, improving the accuracy and reliability of signal analysis.

The db5 wavelet basis function was used to decompose the signal, and then, the low frequency coefficients and high frequency coefficients in the wavelet decomposition structure were extracted. The db5 wavelet is the Daubechies wavelet series (dbN for short). The dbN function is a compactly supported orthonormal wavelet, which makes discrete wavelet analysis possible. Most dbN wavelets do not have symmetry. For orthogonal wavelet functions, the asymmetry is very obvious. Regularity increases with the increase in N. The stronger the normality is, the smoother the wavelet function is, the greater the vanishing moment is, and the wavelet coefficients will be suppressed. Therefore, the db5 wavelet was selected as the wavelet basis function of discrete wavelet transform in this experiment.

Vibration data were decomposed and reconstructed via one-dimensional discrete wavelet transform, and the low-frequency coefficients ca1–ca3 and high-frequency coefficients ch1–ch3 were extracted. Then, the data were processed via two-dimensional discrete wavelet transform, and their reconstruction coefficients a1,v1, and d1 were extracted.

Where ca1–ca3 refers to the high-frequency coefficients in the first to third layers of the one-dimensional wavelet decomposition and ch1–ch3 refers to the low-frequency coefficients in the first to third levels of the wavelet. The 2D reconstructed low-frequency coefficients, vertical directional components of the 2D reconstructed high-frequency coefficients, and diagonal directional components of the 2D reconstructed high-frequency coefficients were designated as a1, v1, and d1, respectively.

**Figure 9:**
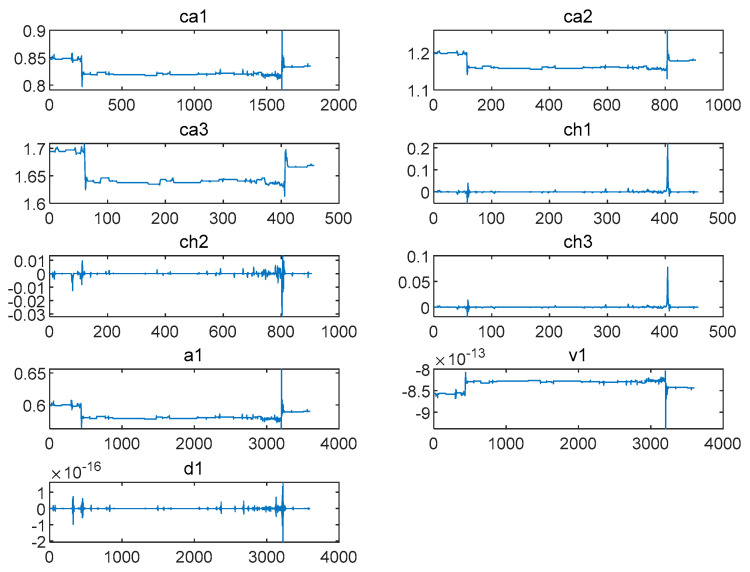
ca1−ca3,ch1−ch3,a1,v1, and d1 images extracted at the inlet during the pumping of Unit 1 (the data of 11A).

As can be seen in Figure 9, the wavelet analysis method was used to denoise, decompose, and reconstruct the pressure fluctuation signals at the inlet and outlet of the draft tube (data 11A) of Unit 1 when pumping water and obtain ca1–ca3,ch1–ch3,a1,v1, and d1.

**Figure 10:**
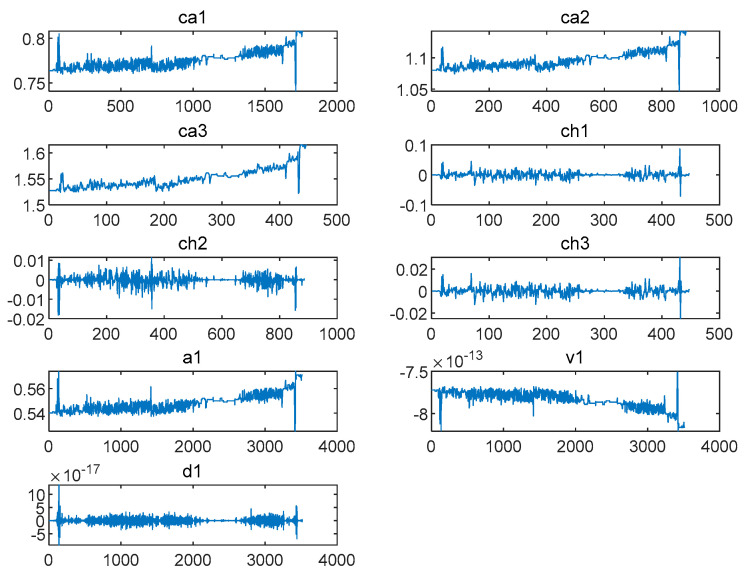
ca1−ca3,ch1−ch3,a1,v1, and d1 images extracted at the inlet during the power generation of Unit 1 (the data of 11B).

As can be seen in Figure 10, the wavelet analysis method was used to denoise, decompose and reconstruct the pressure fluctuation signals at the inlet and outlet of the draft tube (data 11B) of Unit 1 during power generation and obtain ca1–ca3,ch1–ch3,a1,v1, and d1.

**Figure 11:**
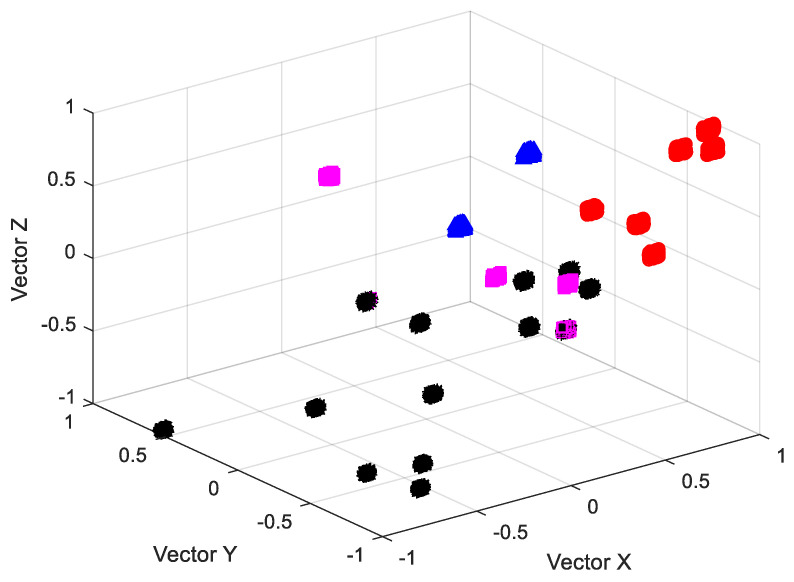
Classification map based on the fuzzy c-means clustering algorithm (FCM).

Fuzzy c-means clustering is one of the most widely used algorithms in fuzzy clustering. According to the similarity between data samples, the fuzzy c-means (FCM) clustering algorithm classifies the samples with high similarity into the same class by iteratively optimizing the objective function. The schematic diagram of the fuzzy c-means (FCM) clustering algorithm classification is shown in Figure 11.

Each set of data contains data from two vibration measuring points at the draft tube inlet and outlet (all measuring points measure the same amount of data in length). The maximum, minimum, sum of squares, and standard deviations of ca1–ca3,ch1–ch3,a1,v1, and d1 are calculated as characteristic vectors, and each group had 36×2 characteristic vectors. Since there are only 24 sets of data in total, the amount of data is too small. Therefore, after 100 repetitions of 24 groups of data, 2400 groups of data are finally obtained, forming a data matrix of 2400 × 72.

Then, the 2400 sets of data are classified as the characteristic vector matrix using the fuzzy c-means (FCM) clustering algorithm. This means using the fuzzy c-means (FCM) clustering algorithm for the initial clustering of pressure fluctuation characteristics of classification and set the number of clustering centers of fuzzy c-means (FCM) clustering algorithm to four, that is, 2400 samples were divided into four categories. The results of classification are as follows: there were 899 samples in the first category, 901 in the second category, 300 in the third category, and 300 in the fourth category.

The fuzzy c-means (FCM) clustering algorithm is used to carry out a random selection of fifty samples from each of the four classification results it processes, for a total of two hundred samples. The first 72 columns of each data sample are the eigenvectors of the sample. The seventy-third column is the output of the classification, represented by the numbers 1, 2, 3, and 4.

### 3.3. Analysis and Comparison of the PNN, the MRFO-PNN and the ITMRFO-PNN Model

The probabilistic neural network (PNN) model is suitable for classification problems. Its strong non-linear classification capability, the requirement to include the most informative characteristic sample of the eigenvectors, and other characteristics make it unique, which applies to the method suggested in Section 2.4.

MATLAB was used to carry out the simulation experiments. The probabilistic neural network (PNN) was used to analyze the 200 data samples selected in Section 3.2. The extracted eigenvectors were used as characteristic fusion input to the probabilistic neural network (PNN), while the output of the probabilistic neural network (PNN) was used to determine the classification of pressure fluctuation. A total of 80 were selected as test samples and 120 were selected as training input samples. Secondly, the probabilistic neural network (PNN) model created had 80 pattern layers, 4 output layers (corresponding to four classification categories), and 120 input layers. If the size of the smoothing factor is too small, the radial basis neurons in the probabilistic neural network will not be able to respond to all the intervals spanned by the input vectors. It will complicate the network calculation if it is too large. As a result, many numbers were manually selected throughout the calculating phase of this experiment to evaluate the categorization effect. The smoothing factor in the probabilistic neural network (PNN) model was selected as 0.9 because the network classification effect performed best when the SPEAD value was 0.9.

**Figure 12:**
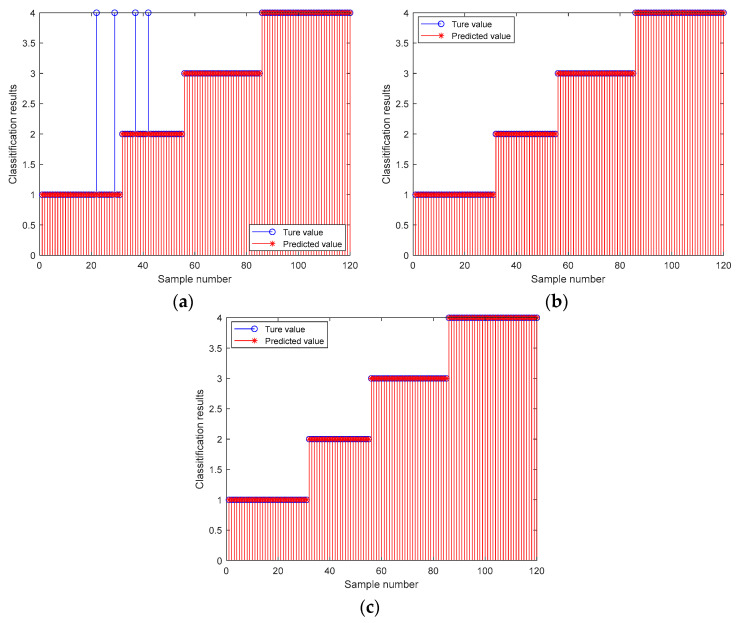
Training diagram of the PNN, MRFO-PNN, and ITMRFO-PNN. (**a**) PNN. (**b**) MRFO-PNN. (**c**) ITMRFO-PNN.

In Figure 12a–c, the horizontal coordinates represent the number of training samples, and the vertical coordinates represent the classification results. The 1–31 sample prediction model belongs to category 1, the 32–55 sample prediction model belongs to category 2, the 56–85 sample prediction model belongs to category 3, and the 86–120 sample prediction model belongs to category 4.

Figure 12a shows the results of probabilistic neural network (PNN) training, where the predicted values of the PNN training set are inconsistent with the true values; four samples were misclassified, and the training accuracy of the probabilistic neural network (PNN) was 96.67%. Figure 12b,c shows the training results for the two models: the optimized probabilistic neural network model based on a manta ray foraging optimization algorithm (MRFO-PNN) and the optimized probabilistic neural network model based on an improved manta ray foraging optimization algorithm (ITMRFO-PNN). With a training accuracy of 100% and accurate predictions, it can be seen that the predicted values of the training samples exactly match the true values.

**Figure 13:**
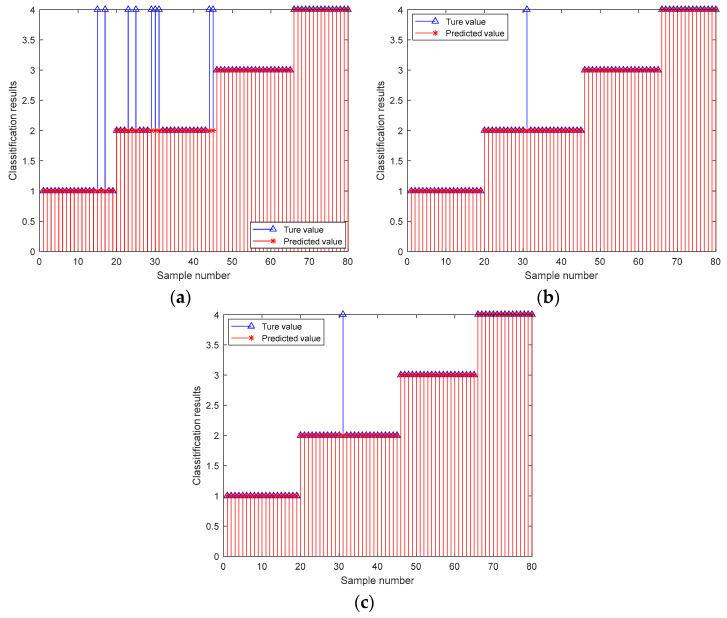
Prediction diagram of the PNN, MRFO-PNN, and ITMRFO-PNN. (**a**) PNN. (**b**) MRFO-PNN. (**c**) ITMRFO-PNN.

In order to further test the extrapolation performance of the three models mentioned above, 120 training samples from the three models in Figure 12 were used to classify and predict the remaining 80 test samples. The results are shown in Figure 13. The prediction patterns for samples 1–19 are category 1, samples 20–45 are category 2, samples 46–65 are category 3, and samples 66–80 are category 4. The horizontal coordinates indicate the test sample numbers, and the vertical coordinates indicate the classification results.

As can be seen in Figure 13a, the predicted values of the probabilistic neural network (PNN) test set were not consistent with the true values, with nine samples mis-classified, and the probabilistic neural network (PNN) test accuracy was 88.75%. From Figure 13b,c, it can be seen that the predicted values of the optimized probabilistic neural net-work model based on a manta ray foraging optimization algorithm (MRFO-PNN) model and optimized probabilistic neural network model based on an improved manta ray foraging optimization algorithm model (ITMRFO-PNN) test set were inconsistent with the true values. Both had one sample misclassification, and both had a test accuracy of 96.67%, indicating high prediction accuracy. At this time, the smoothing factor for the optimized probabilistic neural network model based on a manta ray foraging optimization algorithm model (MRFO-PNN) was 0.31136, and the threshold was 2.674. The smoothing factor and threshold for the optimized probabilistic neural network model based on an improved manta ray foraging optimization algorithm model (ITMRFO-PNN) were 0.30993 and 3.3757, respectively.

Table 10 shows the results of identifying the pressure fluctuation characteristics of a draft tube based on a probabilistic neural network (PNN), an optimized probabilistic neural network model based on a manta ray foraging optimization algorithm model (MRFO-PNN), and an optimized probabilistic neural network model based on an improved manta ray foraging optimization algorithm model (ITMRFO-PNN). From Table 10, it can be concluded that the probabilistic neural network (PNN) model had two identification errors in category 1, seven in category 2, zero in category 3, and zero in category 4. The total identification rate of the model reached 88.75% (71/80). The optimized probabilistic neural network model based on a manta ray foraging optimization algorithm model (MRFO-PNN) and optimized probabilistic neural network model based on an improved manta ray foraging optimization algorithm model (ITMRFO-PNN) both had an identification error in the draft tube pressure fluctuation characteristic of category 2. The total identification rate of both models was 98.75% (79/80). Compared with the non-optimized probabilistic neural network (PNN) model, the identification accuracy of the draft tube pressure fluctuation of the turbine based on an optimized probabilistic neural network model based on a manta ray foraging optimization algorithm model (MRFO-PNN) and an optimized probabilistic neural network model based on an improved manta ray foraging optimization algorithm (ITMRFO-PNN) was extremely high. It can be concluded that both the optimized probabilistic neural network model based on a manta ray foraging optimization algorithm (MRFO-PNN) identification model and the optimized probabilistic neural network model based on an improved manta ray foraging optimization algorithm (ITMRFO-PNN) identification model can effectively identify the draft tube pressure fluctuation of a turbine.

### 3.4. Performance Comparison of the Three Models

We compared the results of our proposed optimized probabilistic neural network model based on an improved manta ray foraging optimization algorithm model (ITMRFO-PNN) with the probabilistic neural network (PNN) model and the optimized probabilistic neural network model based on a manta ray foraging optimization algorithm model (MRFO-PNN) after conducting experiments. Firstly, we will discuss the first performance parameter, which is the confusion matrix.

**Figure 14:**
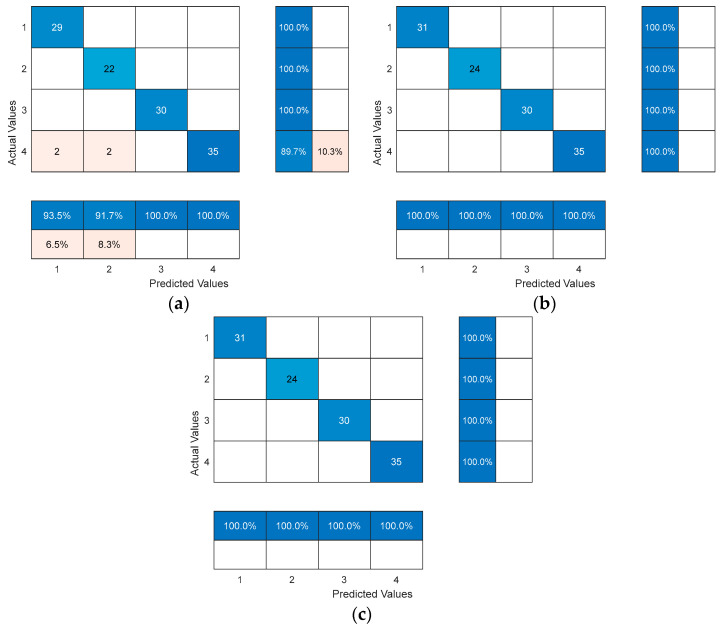
The confusion matrix of the PNN, MRFO-PNN, and ITMRFO-PNN models for the classification of pressure fluctuation signal training sets. (**a**) PNN. (**b**) MRFO-PNN. (**c**) ITMRFO-PNN.

**Figure 15:**
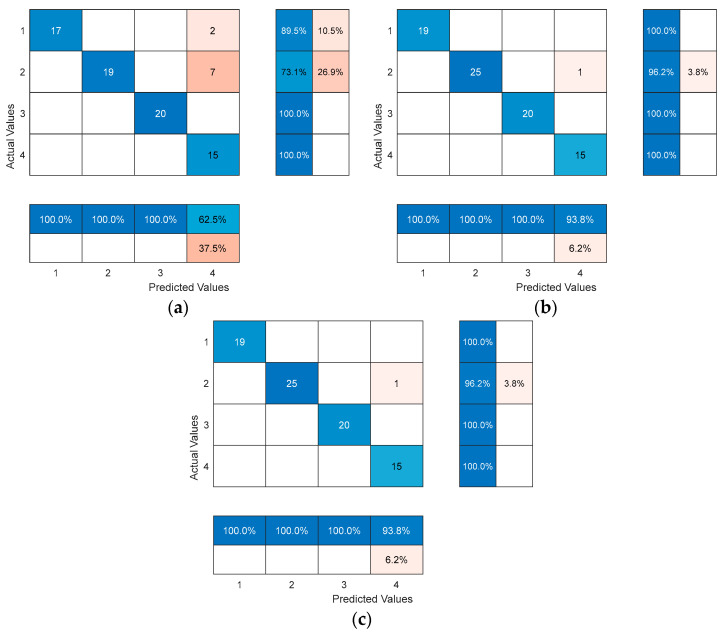
The confusion matrix of the PNN, MRFO-PNN, and ITMRFO-PNN models for the classification of the pressure fluctuation signal test sets. (**a**) PNN. (**b**) MRFO-PNN. (**c**) ITMRFO-PNN.

The confusion matrix plot of the optimized probabilistic neural network model based on an improved manta ray foraging optimization algorithm model (ITMRFO-PNN) for the identification of draft tube pressure fluctuation signals is shown in Figure 14 and Figure 15. The columns represent the actual labeled instances of the classes, while the rows represent the predicted instances of the actual classes. The correct class counts predicted by the model matrix are displayed in the diagonal positions, while the counts of the model’s incorrect predictions are displayed outside the diagonal matrix.

Our proposed model, the optimized probabilistic neural network model based on an improved manta ray foraging optimization algorithm model (ITMRFO-PNN), which is an optimized probabilistic neural network (PNN) model using the improved manta ray foraging optimization algorithm, achieved 100% accuracy in the training samples, as shown in Figure 14. In the testing samples, it achieved a prediction accuracy of 98.7%, as shown in Figure 15.

According to the results in Table 11, our proposed optimized probabilistic neural network model based on an improved manta ray foraging optimization algorithm model (ITMRFO-PNN) exhibited more competitive results compared to the PNN model. The optimized probabilistic neural network model based on a manta ray foraging optimization algorithm model (MRFO-PNN) and optimized probabilistic neural network model based on an improved manta ray foraging optimization algorithm model (ITMRFO-PNN) exhibited 100% in terms of accuracy, precision, recall, and F1-score on the training samples, surpassing the performance of the probabilistic neural network (PNN) model. Furthermore, on the testing samples, the optimized probabilistic neural network model based on a manta ray foraging optimization algorithm model (MRFO-PNN) and optimized probabilistic neural network model based on an improved manta ray foraging optimization algorithm model (ITMRFO-PNN) achieved accuracy of 98.75%, precision of 98.44%, recall of 99.04%, and an impressive F1-score of 98.74% for the classification of pressure fluctuation signals. These results highlight the competitive edge of the optimized probabilistic neural network model based on a manta ray foraging optimization algorithm model (MRFO-PNN) and an optimized probabilistic neural network model based on an improved manta ray foraging optimization algorithm model (ITMRFO-PNN) in accurately and effectively classifying pressure fluctuation signals.

**Figure 16:**
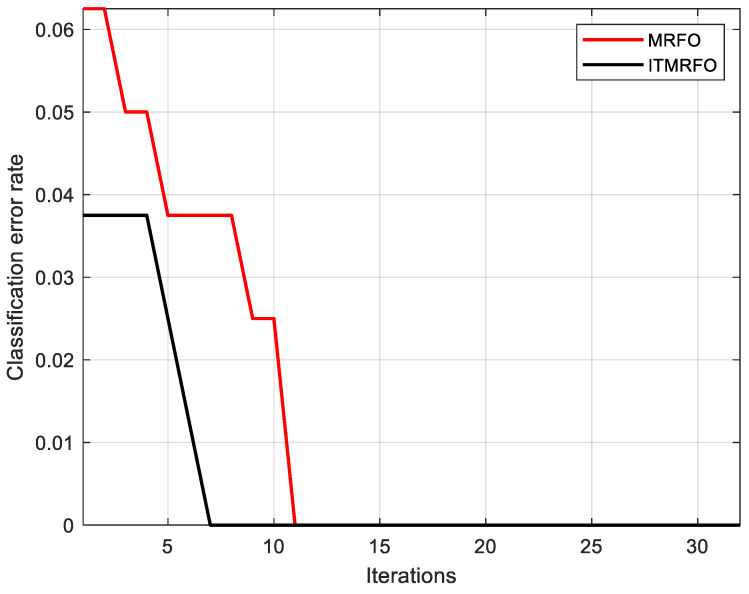
Training error rate of the MRFO-PNN and the ITMRFO-PNN.

Figure 16 shows the training sample identification error rates for the optimized probabilistic neural network model based on a manta ray foraging optimization algorithm model (MRFO-PNN) and the optimized probabilistic neural network model based on an improved manta ray foraging optimization algorithm (ITMRFO-PNN). According to Figure 16 and Table 11, the identification accuracy of the training sample (100%) and the test sample (98.75%) were consistent. However, compared with the optimized probabilistic neural network model based on a manta ray foraging optimization algorithm model (MRFO-PNN) (11 iterations achieved an error rate of zero), the optimized probabilistic neural network model based on an improved manta ray foraging optimization algorithm model (ITMRFO-PNN) achieved an error rate of zero for training sample identification in fewer iterations (only seven iterations achieved an error rate of zero). In addition, the identification error rate of the optimized probabilistic neural network model based on an improved manta ray foraging optimization algorithm model (ITMRFO-PNN) at the beginning of the iteration was less than 0.04, while the identification error rate of the optimized probabilistic neural network model based on a manta ray foraging optimization algorithm model (MRFO-PNN) at the end of the iteration was higher than 0.06. In other words, the identification error rate of the optimized probabilistic neural network model based on an improved manta ray foraging optimization algorithm model (ITMRFO-PNN) at the beginning of iteration was lower than that of the optimized probabilistic neural network model based on a manta ray foraging optimization algorithm model (MRFO-PNN).

## 4. Conclusions

(1) Discrete wavelet transform was used to decompose and reconstruct the collected pressure fluctuation signal, and the maximum, minimum, square sum, and standard deviation of the nine coefficients ca1–ca3,ch1–ch3,a1,v1, and d1 were taken as characteristics. This is a new characteristic extraction method, which provides a new research idea for subsequent data characteristic extraction methods. Following this, the fuzzy c-means (FCM) clustering algorithm was utilized to automatically classify the signal based on its own properties.
(2) Aiming to solve the problem of the manta ray foraging optimization (MRFO) algorithm often falling into the local optimum, the algorithm was improved four times. The elite-opposition-based learning strategy was used to optimize the initial population. The first 50% of the initial population was chosen as the new population to obtain a high-quality population. Adaptive t distribution was used instead of the chain factor to optimize the individual renewal strategy at the chain foraging site. In chain foraging and spiral foraging, the partial expressions for multiplying by r were removed to ensure the stability of the algorithm. In order to evaluate the performance of the improved manta ray foraging optimization (ITMRFO) algorithm, it was compared with three other algorithms including particle swarm optimization (PSO). The results showed that the improved manta ray foraging optimization (ITMRFO) algorithm has high accuracy and efficiency.
(3) To enhance the identification accuracy of the probabilistic neural network (PNN), an improved manta ray foraging optimization (ITMRFO) algorithm was employed to optimize the smoothing factor of the probabilistic neural network, and an ITMRFO-PNN identification model was developed. The model was used to identify the characteristics of pressure fluctuation signals in the draft tube of a hydraulic turbine. The identification results of the probabilistic neural network (PNN), the optimized probabilistic neural network model based on a manta ray foraging optimization algorithm model (MRFO-PNN), and the optimized probabilistic neural network model based on an improved manta ray foraging optimization algorithm model (ITMRFO-PNN) were compared. The identification accuracy for the training samples was 96.67%, 100%, and 100% for the PNN, MRFO-PNN, and ITMRFO-PNN models, respectively. For the test samples, the identification accuracy was 88.75%, 98.75%, and 98.75% for the PNN, MRFO-PNN, and ITMRFO-PNN models, respectively. And we compared these three models through confusion matrix, accuracy, precision, recall, and F1-score. The experimental results show that both MRFO-PNN and ITMRFO-PNN models have better performance than PNN models. However, compared to the MRFO-PNN model (which achieved a zero-bit error rate in eleven iterations), the ITMRFO-PNN model achieved a zero-bit error rate for training sample identification in fewer iterations (only seven iterations achieved a zero-bit error rate). In addition, the identification error rate of the ITMRFO-PNN model at the beginning of the iteration was lower than that of the MRFO-PNN model. Therefore, compared with other algorithms, the improved manta ray foraging optimization (ITMRFO) algorithm has obvious advantages in optimizing a probabilistic neural network (PNN) to identify the pressure fluctuation signal in the draft tube of a hydraulic turbine.

Although the optimized probabilistic neural network model based on an improved manta ray foraging optimization algorithm model (ITMRFO-PNN) demonstrates excellent performance, it also has certain limitations:

(1) The ITMRFO-PNN identification model has much higher identification accuracy than the optimized PNN network model. However, the identification accuracy of this model has not yet reached 100%, and it needs to be improved.
(2) Due to the limitation of the experiment, the amount of data in this study was too small, and using only the pressure fluctuation signal of the Francis turbine draft tube led to poor comparability of the data. This also means that data from other types of hydraulic turbine units cannot be verified, and further verification is needed for state identification of other types.

Therefore, future research can select additional types of sample data and conduct further in-depth research based on the optimized probabilistic neural network model based on the improved manta ray foraging optimization algorithm model (ITMRFO-PNN) with the feature identification methods proposed in this study.

## Figures and Tables

**Table 1:** Algorithm parameter.

Algorithms	Main Parameters
SSA	Early warning value ST=0.6, the weight of finder ST=0.7, the weight of joiner ST=0.3, and Sparrows aware of danger weight ST=0.2
PSO	ω=0.9, $c_{1}=c_{2}=2$
MRFO	S=2
ITMRFO	S=2

**Table 2:** Unimodal test functions.

Function	D	Range
$F_{1}(X)=\sum_{i=1}^{n}x_{i}^{2}$	30	${\left[-100,100\right]}^{D}$
$F_{2}(X)=\sum_{i=1}^{n}\left|x_{i}\right|+\prod_{i=1}^{n}\left|x_{i}\right|$	30	${\left[-10,10\right]}^{D}$
$F_{3}(X)=\sum_{i=1}^{n}{\left(\sum_{j=1}^{n}x_{j}\right)}^{2}$	30	${\left[-100,100\right]}^{D}$
$F_{4}(X)=max\left\{\left|x_{i}\right|,1\leq i\leq n\right\}$	30	${\left[-100,100\right]}^{D}$
$F_{5}(X)=\sum_{i=1}^{n}\left[100{\left(x_{i+1}-x_{i}^{2}\right)}^{2}+{\left(x_{i}-1\right)}^{2}\right]$	30	${\left[-30,30\right]}^{D}$
$F_{6}(X)=\sum_{i=1}^{n-1}{\left(\left[x_{i}+0.5\right]\right)}^{2}$	30	${\left[-100,100\right]}^{D}$
$F_{7}(X)=\sum_{i=1}^{n}{ix}_{i}^{4}+random[0,1)$	30	${\left[-1.28,1.28\right]}^{D}$

**Table 3:** Multimodal test functions.

Function	D	Range
$F_{8}(X)=\sum_{i=1}^{n}-x_{i}\sin\left(\sqrt{\left|x_{i}\right|}\right)$	30	${\left[-500,500\right]}^{D}$
$F_{9}(X)=\sum_{i=1}^{n}\left[x_{i}^{2}-10\cos(2\pi x_{i})+10\right]$	30	${\left[-5.12,5.12\right]}^{D}$
$F_{10}(X)=-20exp(-0.2\sqrt{\frac{1}{n}\sum_{i=1}^{n}x_{i}^{2}})-exp(\frac{1}{n}\sum_{i=1}^{n}\cos\left(2\pi x_{i}\right))\;\;\;\;\;\;\;+\;20+e$	30	${\left[-32,32\right]}^{D}$
$F_{11}(X)=\frac{1}{4000}\sum_{i=1}^{n}x_{i}^{2}-\prod_{i=1}^{n}\cos\frac{x_{i}}{\sqrt{i}}+1$	30	${\left[-600,600\right]}^{D}$
$F_{12}\left(x\right)=\frac{\pi }{n}\left\{10{sin}^{2}\left(\pi y_{1}\right)\;\;\;\;\;\;\;+\sum_{i=1}^{n-1}{{(y}_{1}-1)}^{2}\left[1+10{sin}^{2}\left(\pi y_{1}+1\right)\right]\;\;\;\;\;\;\;+\;({y_{n}-1)}^{2}\right\}+\sum_{i=1}^{30}u(x_{i},10,100,4)\;\;\;\;\;\;\;+\sum_{i=1}^{n}u(x_{i},10,100,4)$	30	${\left[-50,50\right]}^{D}$
$F_{13}\left(x\right)=0.1\left\{{sin}^{2}\left(3\pi x_{1}\right)\;\;\;\;\;\;\;+\sum_{i=1}^{29}{{(x}_{i}-1)}^{2}p\left[1+10{sin}^{2}\left(3\pi x_{i+1}\right)\right]\;\;\;\;\;\;\;+\;{{(x}_{n}-1)}^{2}[1+{sin}^{2}\left(2\pi x_{30}\right)]\right\}\;\;\;\;\;\;\;+\sum_{i=1}^{30}u(x_{i},5,10,4)$	30	${\left[-50,50\right]}^{D}$

**Table 4:** Fixed-dimensional multimodal test functions.

Function	D	Range
$F_{14}(X)={\left(\frac{1}{500}+\sum_{j=1}^{25}\frac{1}{j\;+\;\sum_{i=1}^{2}{\left({x_{i}\;-\;a}_{ij}\right)}^{6}}\right)}^{-1}$	2	${\left[-65.536,65.536\right]}^{D}$
$F_{15}(X)=\sum_{i=1}^{11}{\left[a_{i}-\frac{x_{1}\left(b_{i}^{2}\;+\;b_{i}x_{2}\right)}{b_{i}^{2}\;+\;b_{i}x_{3}\;+\;x_{4}}\right]}^{2}$	4	${\left[-5,5\right]}^{D}$
$F_{16}\left(X\right)=4x_{1}^{2}-2.1x_{1}^{4}+\frac{1}{3}x_{1}^{6}+x_{1}x_{2}-4x_{2}^{2}{\;+\;4x}_{2}^{4}$	2	${\left[-5,5\right]}^{D}$
$F_{17}\left(X\right)=-{\left(x_{2}-\frac{5.1}{4\pi^{2}}x_{1}^{2}+\frac{5}{\pi }x_{1}-6\right)}^{2}+10\left(1-\frac{1}{8\pi }\right)\cos x_{1}+10$	2	$\left[-5,10\right]\times [0,15]$
$F_{18}\left(X\right)=\left[1+{\left(x_{1}+x_{2}+1\right)}^{2}{\left(19-14x_{1}+3x_{1}^{2}-14x_{2}+6x_{1}x_{2}\;\;\;\;\;\;\;\;+\;3x_{2}^{2}\right)}^{2}\;\;\;\;\;\;\;\;\times \left(18-32x_{1}+12x_{1}^{2}+48x_{2}-36x_{1}x_{2}\;\;\;\;\;\;\;\;+\;27x_{2}^{2}\right)\right]$	2	${\left[-2,2\right]}^{D}$
$F_{19}\left(X\right)=-\sum_{i=1}^{4}c_{i}exp\left(-\sum_{j=1}^{3}a_{ij}{\left(x_{j}-p_{ij}\right)}^{2}\right)$	3	${\left[0,1\right]}^{D}$
$F_{20}\left(X\right)=-\sum_{i=1}^{4}c_{i}exp\left(-\sum_{j=1}^{6}a_{ij}{\left(x_{j}-p_{ij}\right)}^{2}\right)$	6	${\left[0,1\right]}^{D}$
$F_{21}\left(X\right)=-\sum_{i=1}^{5}{\left[\left(X-a_{i}\right){\left(X-a_{i}\right)}^{T}+c_{i}\right]}^{-1}$	4	${\left[0,10\right]}^{D}$
$F_{22}\left(X\right)=-\sum_{i=1}^{7}{\left[\left(X-a_{i}\right){\left(X-a_{i}\right)}^{T}+c_{i}\right]}^{-1}$	4	${\left[0,10\right]}^{D}$
$F_{23}\left(X\right)=-\sum_{i=1}^{10}{\left[\left(X-a_{i}\right){\left(X-a_{i}\right)}^{T}+c_{i}\right]}^{-1}$	4	${\left[0,10\right]}^{D}$

**Table 5:** Optimization results of the four intelligent optimization algorithms on unimodal test functions.

Function	Value	SSA	PSO	MRFO	ITMRFO
**F1**	**Optimum**	**0**	2.2590 × 10	**0**	**0**
	**Worst**	1.1305 × 10^42^	6.5671 × 10^2^	**0**	**0**
	**Mean**	3.7683 × 10^44^	3.2095 × 10^2^	**0**	**0**
	**Standard**	2.064 × 10^43^	1.6547 × 10^2^	**0**	**0**
**F2**	**Optimum**	**0**	4.096	2.1756 × 10	**0**
	**Worst**	4.6943 × 10^−18^	3.5087 × 10	8.5057 × 10^−202^	**1.1006 × 10^−289^**
	**Mean**	1.5648 × 10^−19^	1.6695 × 10	2.8356 × 10^−203^	**6.4115 × 10^−291^**
	**Standard**	8.5706 × 10^−19^	7.1839	**0**	**0**
**F3**	**Optimum**	**0**	1.2559 × 10^3^	**0**	**0**
	**Worst**	8.5409 × 10^−31^	2.7754 × 10^4^	**0**	**0**
	**Mean**	2.847 × 10^−32^	7.8126 × 10^3^	**0**	**0**
	**Standard**	1.5594 × 10^−31^	5.8392 × 10^3^	**0**	**0**
**F4**	**Optimum**	**0**	6.6225	1.3387 × 10^−211^	1.0118 × 10^−310^
	**Worst**	1.2899 × 10^−40^	1.4551 × 10	7.9795 × 10^−202^	**5.6387 × 10^−284^**
	**Mean**	4.2997 × 10^−42^	1.0199 × 10	6.1482 × 10^−203^	**1.8796 × 10^−285^**
	**Standard**	2.355 × 10	2.3013	**0**	**0**
**F5**	**Optimum**	**5.5425 × 10^−6^**	4.2109 × 10^2^	2.1162 × 10	2.7003 × 10
	**Worst**	**7.3554 × 10^−2^**	3.9617 × 10^4^	2.3790 × 10	2.8860 × 10
	**Mean**	**2.0988 × 10^−2^**	1.2007 × 10^4^	2.2765 × 10	2.8152 × 10
	**Standard**	**1.6765 × 10^−2^**	8.7865 × 10^3^	5.6801 × 10^−1^	6.9318 × 10^−1^
**F6**	**Optimum**	1.9537 × 10^−5^	6.7162 × 10	**4.7686 × 10^−12^**	1.9852
	**Worst**	2.5253 × 10^−4^	6.3960 × 10	**8.6445 × 10^−10^**	3.8045
	**Mean**	8.6289 × 10^−5^	3.4391 × 10^2^	**9.3891 × 10^−11^**	2.9016
	**Standard**	5.0853 × 10^−5^	1.2485 × 10^2^	**1.5542 × 10^−10^**	4.32 × 10^−1^
**F7**	**Optimum**	1.9284 × 10^−4^	4.2776 × 10^−2^	7.7868 × 10^−6^	**3.7422 × 10^−6^**
	**Worst**	1.7391 × 10^−3^	1.8851 × 10	4.6089 × 10^−4^	**3.5646 × 10^−4^**
	**Mean**	6.4509 × 10^−4^	1.928	1.3824 × 10^−4^	**7.7893 × 10^−5^**
	**Standard**	4.2451 × 10^−4^	4.8185	1.1841 × 10^−4^	**8.6124 × 10^−5^**

**Table 6:** Optimization results of the four intelligent optimization algorithms on multimodal test functions.

Function	Value	SSA	PSO	MRFO	ITMRFO
**F8**	**Optimum**	−1.2569 × 10^4^	−9.8631 × 10^3^	−9.3716 × 10^3^	**−6.9461 × 10^16^**
	**Worst**	−5.2284 × 10^3^	−5.4187 × 10^3^	**−6.9232 × 10^3^**	−4.6265 × 10^3^
	**Mean**	−8.8956 × 10^3^	−7.5328 × 10^3^	−8.3555 × 10^3^	**−2.3154 × 10^15^**
	**Standard**	2.5607 × 10^3^	1.0890 × 10^3^	**6.2578 × 10^2^**	1.2682 × 10
**F9**	**Optimum**	**0**	1.5453 × 10^2^	**0**	**0**
	**Worst**	**0**	2.5812 × 10^5^	**0**	**0**
	**Mean**	**0**	1.9755 × 10^5^	**0**	**0**
	**Standard**	**0**	3.0430 × 10	**0**	**0**
**F10**	**Optimum**	**8.8818 × 10^−16^**	4.3941	**8.8818 × 10^−16^**	**8.8818 × 10^−16^**
	**Worst**	**8.8818 × 10^−16^**	7.4033	**8.8818 × 10^−16^**	**8.8818 × 10^−16^**
	**Mean**	**8.8818 × 10^−16^**	5.8133	**8.8818 × 10^−16^**	**8.8818 × 10^−16^**
	**Standard**	**0**	9.2016 × 10^−1^	**0**	**0**
**F11**	**Optimum**	**0**	1.9237	**0**	**0**
	**Worst**	**0**	7.8588	**0**	**0**
	**Mean**	**0**	4.0544	**0**	**0**
	**Standard**	**0**	1.5093	**0**	**0**
**F12**	**Optimum**	2.9046 × 10^−6^	7.563 × 10^−1^	**1.2186 × 10^−13^**	1.1021 × 10^−1^
	**Worst**	6.2883 × 10^−5^	1.5219 × 10	**8.7152 × 10^−12^**	4.0626 × 10^−1^
	**Mean**	2.1853 × 10^−5^	6.6329	**2.7254 × 10^−12^**	2.3181 × 10^−1^
	**Standard**	1.622 × 10^−5^	3.1326	**2.4554 × 10^−12^**	6.2942 × 10^−2^
**F13**	**Optimum**	**4.5633 × 10^−6^**	4.9391	1.0987 × 10^−2^	1.6503
	**Worst**	**4.693 × 10^−2^**	3.9108 × 10	2.9661	2.9842
	**Mean**	**5.1678 × 10^−3^**	2.0094 × 10	2.6196	2.7706
	**Standard**	**9.7266 × 10^−3^**	8.9844	9.1335 × 10^−1^	4.1936 × 10^−1^

**Table 7:** Optimization results of the four optimization algorithms for fixed-dimensional multimodal test functions.

Function	Value	SSA	PSO	MRFO	ITMRFO
**F14**	**Optimum**	**9.98 × 10^−1^**	**9.98 × 10^−1^**	**9.98 × 10^−1^**	**9.98 × 10^−1^**
	**Worst**	1.2671 × 10	**9.98 × 10^−1^**	**9.98 × 10^−1^**	2.9821
	**Mean**	1.02478 × 10	**9.98 × 10^−1^**	**9.98 × 10^−1^**	1.1964
	**Standard**	4.299	1.698 × 10^−10^	**5.8312 × 10^−17^**	6.0541 × 10^−1^
**F15**	**Optimum**	3.0849 × 10^−4^	6.4758 × 10^−4^	3.0749 × 10^−4^	**3.0749 × 10^−4^**
	**Worst**	**4.8808 × 10^−4^**	2.973 × 10^−2^	1.2232 × 10^−4^	7.−04 × 10^−4^
	**Mean**	**3.3932 × 10^−4^**	1.3029 × 10^−2^	4.2958 × 10^−4^	3.444 × 10^−4^
	**Standard**	**3.9562 × 10^−5^**	1.0006 × 10^−2^	3.166 × 10^−4^	1.1039 × 10^−4^
**F16**	**Optimum**	**−1.0316**	**−1.0316**	**−1.0316**	**−1.0316**
	**Worst**	**−1.0316**	**−1.0316**	**−1.0316**	**−1.0316**
	**Mean**	**−1.0316**	**−1.0316**	**−1.0316**	**−1.0316**
	**Standard**	8.6536 × 10^−8^	7.299 × 10^−5^	6.5843 × 10^−16^	**6.1158 × 10^−16^**
**F17**	**Optimum**	**3.9789 × 10^−1^**	**3.9789 × 10^−1^**	**3.9789 × 10^−1^**	**3.9789 × 10^−1^**
	**Worst**	**3.9789 × 10^−1^**	**3.9789 × 10^−1^**	**3.9789 × 10^−1^**	**3.9789 × 10^−1^**
	**Mean**	**3.9789 × 10^−1^**	**3.9789 × 10^−1^**	**3.9789 × 10^−1^**	**3.9789 × 10^−1^**
	**Standard**	4.9682 × 10^−7^	1.6488 × 10^−5^	**0**	**0**
**F18**	**Optimum**	**3**	**3**	**3**	**3**
	**Worst**	**3**	3.0017	**3**	**3**
	**Mean**	**3**	3.0002	**3**	**3**
	**Standard**	2.1119 × 10^−6^	3.5805 × 10^−4^	1.686 × 10^−15^	**1.3946 × 10^−15^**
**F19**	**Optimum**	**−3.8628**	**−3.8628**	**−3.8628**	**−3.8628**
	**Worst**	**−3.8628**	**−3.8628**	**−3.8628**	**−3.8628**
	**Mean**	**−3.8628**	**−3.8628**	**−3.8628**	**−3.8628**
	**Standard**	3.6072 × 10^−5^	3.7109 × 10^−3^	**2.6543 × 10^−15^**	1.4074 × 10^−13^
**F20**	**Optimum**	**−3.322**	−3.3215	**−3.322**	−3.3219
	**Worst**	−3.1149	−2.7015	**−3.2031**	−3.1144
	**Mean**	−3.2519	−3.1	−3.2586	**−3.2683**
	**Standard**	7.7881 × 10^−2^	1.9408 × 10^−1^	**6.0328 × 10^−2^**	6.1883 × 10^−2^
**F21**	**Optimum**	**−1.0153 × 10**	**−1.0153 × 10**	**−1.0153 × 10^−1^**	**−1.0153× 10**
	**Worst**	**−1.0151 × 10**	−2.6284	−5.0552	−5.0368
	**Mean**	**−1.01525 × 10**	−8.1955	−7.9441	−8.383
	**Standard**	**5.597 × 10^−4^**	2.6096	2.5694	2.4039
**F22**	**Optimum**	**−1.0403 × 10**	**−1.0403 × 10**	**−1.0403 × 10**	**−1.0403 × 10**
	**Worst**	**−1.0402 × 10**	−2.7615	−3.7243	−3.7077
	**Mean**	**−1.0403 × 10**	−9.6874	−8.7629	−8.2068
	**Standard**	**2.4731 × 10^−4^**	1.873	2.5592	2.6558
**F23**	**Optimum**	**−1.0536 × 10**	**−1.0536 × 10**	**−1.0536 × 10**	**−1.0536 × 10**
	**Worst**	**−1.0535 × 10**	−1.0306 × 10	−3.8354	−5.0093
	**Mean**	**−1.0536 × 10**	−1.0487 × 10	−9.0512	−9.3896
	**Standard**	**2.5315 × 10^−4^**	6.4829 × 10^−2^	2.5151	2.0902

**Table 8:** The twenty-four groups of test data.

Unit 1		Unit 2		Unit 3		Unit 4	
W1	P1	W2	P2	W3	P3	W4	P4
11A	11B	21A	21B	31A	31B	41A	41B
12A	12B	22A	22B	32A	32B	42A	42B
13A	13B	23A	23B	33A	33B	43A	43B

**Table 9:** Partial pressure fluctuation data of 11A.

Serial Number	Import	Export
1	0.561	0.601
2	0.561	0.601
3	0.561	0.601
4	0.561	0.601
5	0.561	0.601
6	0.561	0.601
7	0.561	0.601
8	0.561	0.601
9	0.561	0.601
10	0.561	0.601
11	0.561	0.601
12	0.561	0.601
13	0.561	0.601
14	0.561	0.601
15	0.561	0.601

**Table 10:** Comparison of accuracy, precision, recall, and F1-score of the three models.

Model	Category 1	Category 2	Category 3	Category 4	Accuracy %
PNN	17/19	19/26	20/20	15/15	88.75 (71/80)
MRFO-PNN	19/19	25/26	20/20	15/15	98.75 (79/80)
ITMRFO-PNN	19/19	25/26	20/20	15/15	98.75 (79/80)

**Table 11:** Comparison of the characteristic identification results of the three models.

Model	Training/Testing Results	Accuracy %	Precision	Recall	F1-Score
PNN	Training	96.67	96.30	97.43	96.87
	Testing	88.75	92.63	90.64	90.63
MRFO-PNN	Training	100	100	100	100
	Testing	98.75	98.44	99.04	98.74
ITMRFO-PNN	Training	100	100	100	100
	Testing	98.75	98.44	99.04	98.74

## Data Availability

The numerical and experimental data used to support the findings of this study are included within the article.
